# FmMDb: A Versatile Database of Foxtail Millet Markers for Millets and Bioenergy Grasses Research

**DOI:** 10.1371/journal.pone.0071418

**Published:** 2013-08-12

**Authors:** Venkata Suresh B, Mehanathan Muthamilarasan, Gopal Misra, Manoj Prasad

**Affiliations:** National Institute of Plant Genome Research, New Delhi, India; University of Lausanne, Switzerland

## Abstract

The prominent attributes of foxtail millet (*Setaria italica* L.) including its small genome size, short life cycle, inbreeding nature, and phylogenetic proximity to various biofuel crops have made this crop an excellent model system to investigate various aspects of architectural, evolutionary and physiological significances in Panicoid bioenergy grasses. After release of its whole genome sequence, large-scale genomic resources in terms of molecular markers were generated for the improvement of both foxtail millet and its related species. Hence it is now essential to congregate, curate and make available these genomic resources for the benefit of researchers and breeders working towards crop improvement. In view of this, we have constructed the Foxtail millet Marker Database (FmMDb; http://www.nipgr.res.in/foxtail.html), a comprehensive online database for information retrieval, visualization and management of large-scale marker datasets with unrestricted public access. FmMDb is the first database which provides complete marker information to the plant science community attempting to produce elite cultivars of millet and bioenergy grass species, thus addressing global food insecurity.

## Introduction

Foxtail millet [*Setaria italica* (L.) P. Beauv.] is the second most largely cultivated millet. Its potential abiotic stress tolerance and its genetic relatedness to several bioenergy grasses such as switchgrass (*Panicum virgatum*), napier grass (*Pennisetum purpureum*) and pearl millet (*Pennisetum glaucum*) has made this crop a tractable experimental model for studying functional genomics of millets and bioenergy crops [1], [2]. Further, foxtail millet is an economically important crop grown and consumed all over the world, especially in India, China, and other parts of Asia, North Africa, and the Americas. Hence, considering the prominence of foxtail millet as a model crop, the US Department of Energy - Joint Genome Institute and the Beijing Genomics Institute, China have sequenced its genome, and the sequence was released in 2012 [3], [4]. The availability of the genome sequence motivated the millet research community to decipher and analyse the genomic and genetic data, which eventually resulted in the generation of large-scale genomic-, genic-simple sequence repeats (SSRs) and intron length polymorphic (ILP) markers, as well as the development of physical maps [5]–[9]. Noteworthy, all the experimental outcomes showed a high percentage of marker cross-transferability in several millets and bioenergy grass species. This suggests that these markers can be useful in various large-scale genotyping applications including germplasm characterization, cultivar identification, construction of high-density microsatellite marker-based physical map for gene/QTL discovery and comparative genome mapping involving foxtail millet and other millets, cereals and bioenergy grass species [5]–[9].

With the availability of novel, large-scale analyzed data on molecular markers, it became necessary to establish a unique database in order to facilitate unrestricted access for breeders and researchers to these genomic resources, and facilitate their use in genetic improvement of target millet and bioenergy grass species. In order to fulfil this need of the global millets and bioenergy grasses research community, the Foxtail millet Marker Database (FmMDb; http://www.nipgr.res.in/foxtail.html) has been constructed. No such database had been developed till date for markers in *Setaria* sp. or related biofuel crops, and hence FmMDb is the first comprehensive database dedicated for structural and comparative genomics in millet and bioenergy grass species.

## Methods

### Database Architecture

**Figure 1:**
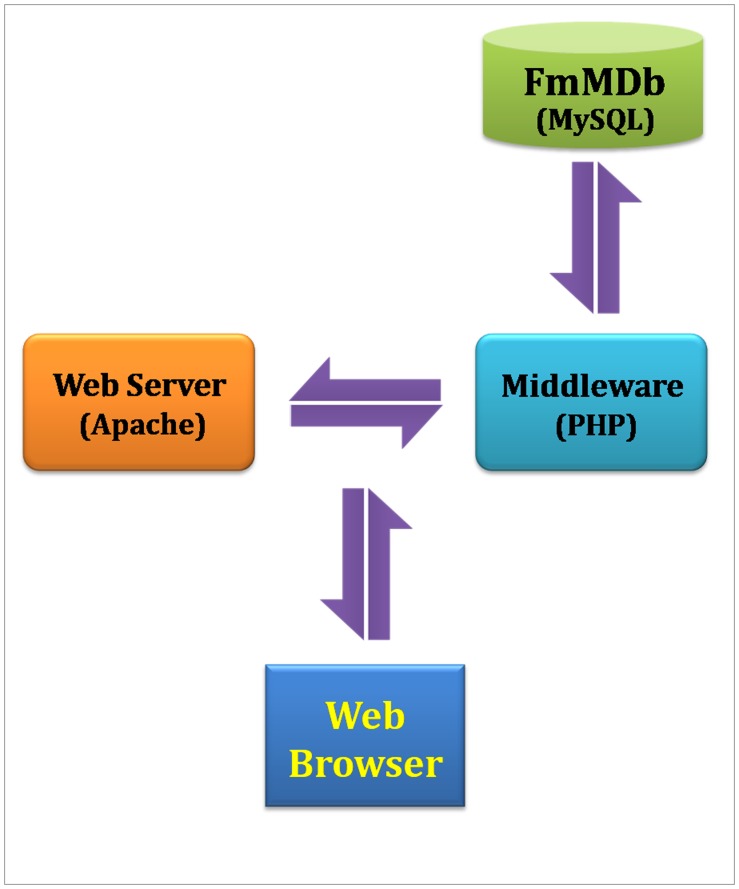
Three-level schema architecture of FmMDb.

**Figure 2:**
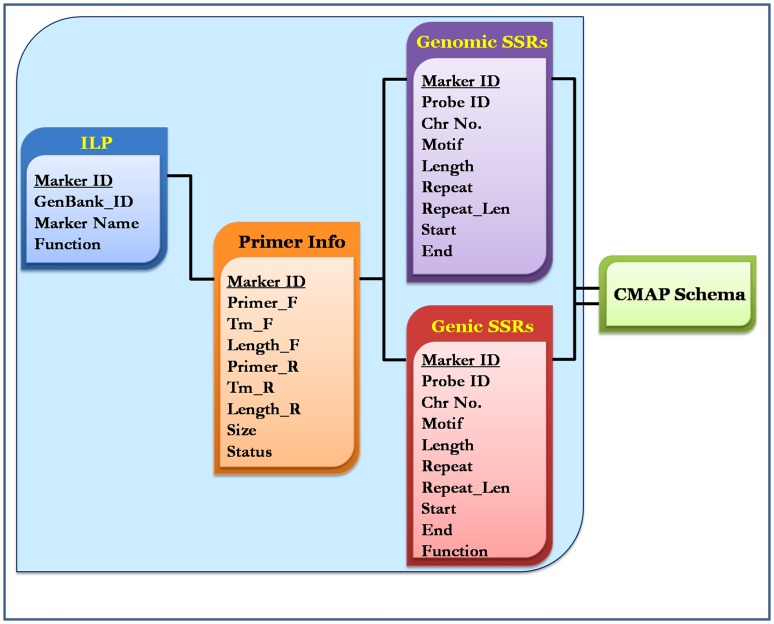
Database schema.

The Foxtail millet Marker Database is an online interactive searchable and downloadable database developed using MySQL 5.0 (www.mysql.com), and it serves as a repository for three kinds of DNA marker data, namely genomic SSRs [5]–[7], genic SSRs [8] and ILP markers [9]. The database was designed based on ‘Three-Level Schema Architecture’ (Figure 1). The CMap schema [10] has been integrated with FmMDb for facilitating interactive visualization of physical map and comparative mapping of the marker data with genomes of agronomically important crop species such as *Sorghum bicolor, Zea mays, Oryza sativa* and *Brachypodium distachyon*. The comprehensive schema of FmMDb is shown in Figure 2. Currently, the database encompasses the information of 21,315 genomic SSRs, 447 genic SSRs and 96 ILP markers. In addition, ‘Microsatellite Repeat Finder’ interface is also mirrored by the database for detecting microsatellites in any given query nucleotide sequence. The code was retrieved from BioPHP - Microsatellite Repeats Finder (http://www.biophp.org/minitools/microsatellite_repeats_finder/). The BioPHP (recursive acronym: Hypertext Preprocessor scripts for Biology) version 2.0 is an open source project which offers a range of PHP scripts for bio-computational analyses. The script was modified to accept sequences greater than 100 kb as query, and the parameter “Allowed percentage of mismatches” was removed in order to ensure the accuracy of output.

### User Interface Utility

**Figure 3:**
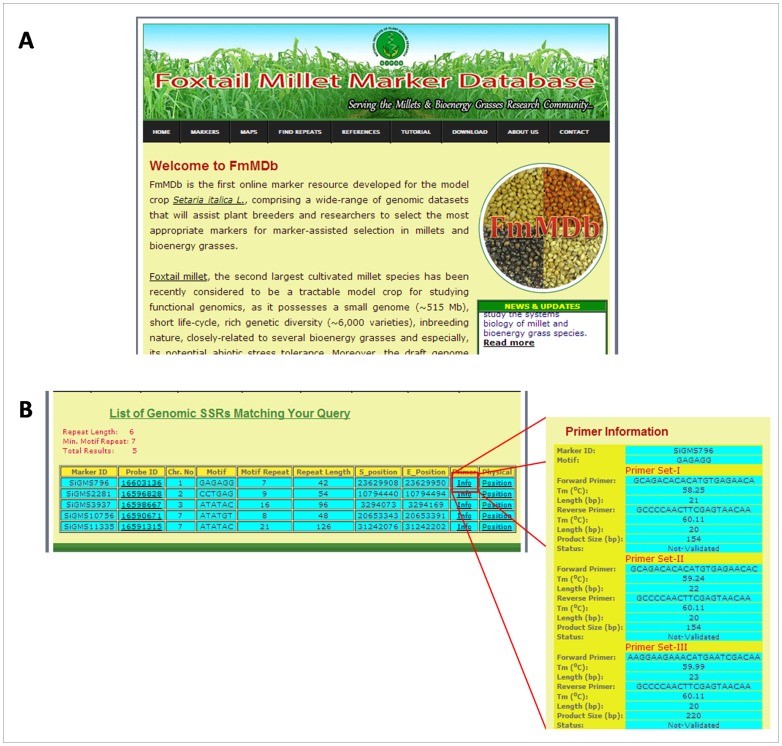
Screenshots of FmMDb (A) Homepage displaying the different options available for the user (B) A typical search result for genomic SSR query; the ‘Info’ displays the information on primers.

The user-friendly interface for FmMDb was developed using PHP 5.4 (www.php.net) and HTML for query input and retrieval of data as per users’ requirement (Figure 3). The database provides different search parameters for the user to choose the desired marker based on motif sequence of marker, desired marker repeat length and minimum number of repeats, NCBI probe ID, chromosome number or the function (in case of genic SSRs and ILPs). The result for the respective query will be tabulated with Marker ID, Motif details (sequence, length, repeat type) repeat length, chromosomal location, start and end positions, functional information (in case of genic SSRs) along with hyperlinks to primer information (primer ‘Info’) and physical map (physical ‘Position’) (Figure 3).

**Figure 4:**
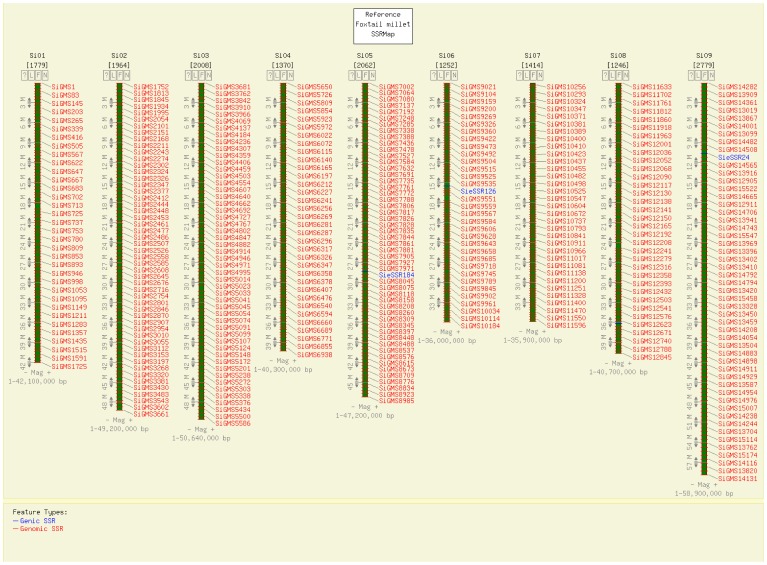
Physical map of the foxtail millet generated by the CMap interface. The segments of nine chromosomes mapped with SSRs (in red) and eSSRs (is blue) is shown. Individual marker details can be viewed by clicking onto the respective marker ID.

**Figure 5:**
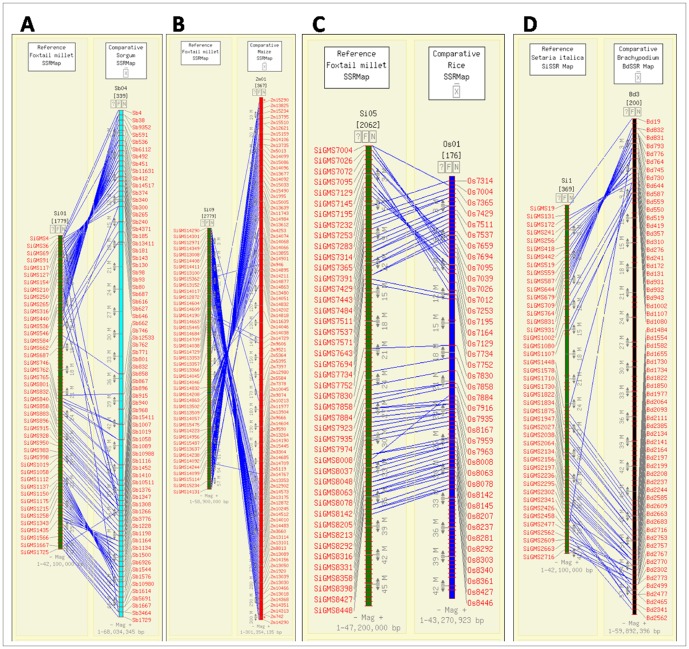
A segment of comparative map displayed through CMap interface showing the syntenic correspondences (in blue lines) between (A) foxtail millet chromosome 1 and sorghum chromosome 4, (B) foxtail millet chromosome 9 and maize chromosome 1, (C) foxtail millet chromosome 5 and rice chromosome 1, (D) foxtail millet chromosome 1 and *Brachypodium* chromosome 3. The foxtail millet chromosomes showing maximum synteny [Ref: 5,8] with the chromosomes of sorghum, maize, rice and *Brachypodium* was shown.

The ‘Info’ hyperlink under primer redirects to the primer information page, which lists details of the respective primer. The details provided under ‘Primer Information’ include forward and reverse primer sequences (a maximum of three primer pairs will be displayed), respective melting temperatures (Tm), lengths, expected product size and the status on primer validation. The physical map hyperlink directs to CMap interface, where the user can view the physical locations of the markers on the foxtail millet genome (Figure 4). CMap also allows the user to visualize the syntenic relationships based on comparative genome mapping between the chromosomes of foxtail millet and sorghum, maize, rice and *Brachypodium* (Figure 5).

The ‘Microsatellite Repeat Finder’ tool assists the user in detecting the repeats present in a given nucleotide query sequence. It has three adjustable search parameters, namely (i) motif length (minimum and maximum), (ii) minimum number of repeats to be identified and (iii) minimum number of tandem repeats to be detected. The tool will search the query for repeat sequences which conforms user requirements and generate the result. The output will display the start position of the repeat, its motif length, repeat length and the motif sequence.

Finally, the complete marker data of FmMDb can be downloaded from the ‘Download’ section for the user’s perusal. Moreover, a ‘Tutorial’ has also been provided to increase the usability.

## Results and Discussion

Although plant breeding is principally based on phenotypic selection of elite individuals amongst segregating progenies resulting from hybridization, molecular markers play a crucial role in breeding through both genotypic and phenotypic selection. Marker-assisted selection (MAS) implicates the selection of plants possessing genomic regions that are involved in the expression of agronomically important traits and thus, with the development and accessibility of an array of DNA markers and dense molecular maps, MAS appears to be promising for traits governed by both major genes and quantitative trait loci (QTLs) [11], [12]. Hence DNA markers are unambiguously implemented in various structural, functional and comparative genomic applications including variety identification, studying genetic diversity and phylogenetic relationships, construction of high density genome maps, mapping of useful genes and comparative genome mapping. This has resulted in the generation of large-scale DNA markers in a variety of crop plants, and has subsequently led to the development of relevant marker databases for the benefit of both plant breeders and researchers [13]–[20]. But, till date no such database is available for foxtail millet or any bioenergy grass species although the crop has many salient characteristics such as: (i) small genome size (∼515 Mb; 2n = 2x = 18) with a relatively lower amount of repetitive DNA [1]–[4], (ii) inbreeding nature coupled to short life-cycle [1]–[4], (iii) genetically related to several biofuel grasses with complex genomes [1], (iv) potential abiotic stress tolerance, in particular for drought and salinity [11], [21]–[27], (v) rich genetic diversity (∼6,000 varieties) and existence of a complete collection of germplasm [28], (vi) availability of high-throughput transformation platforms [1], [28]–[34], (vii) availability of draft genome sequence [3], [4], and (viii) high percentage of cross-genera transferability of DNA markers to other millets, bioenergy grasses and non-millet crops [5]–[9]. These merits have made the crop a tractable experimental model system for studying the functional genomics of millets and bioenergy crops [1]. Hence this marker database serves as a repository of DNA markers of a model plant, assisting the millets and bioenergy grasses research community. The notable features of FmMDb include;

- User-friendly search criteria for easy retrieval of marker data.
- Comprehensive notes and figures summarizing the strategies employed for generating the markers and the respective outcomes has been provided for the knowledge of users.
- Direct hyperlinking of NCBI IDs to respective submission page in NCBI database has been facilitated.
- For each microsatellite marker, the database provides three primer pairs along with all the relevant information of the primers (Figure 3). This provides an option to the users in choosing the apt primer pair for their downstream experimentations.
- An interactive physical and comparative map was developed using the CMap interface, thus facilitating the localisation of the position of a marker on the foxtail millet chromosomes along with its corresponding position on the chromosomes of sorghum, maize, rice and *Brachypodium* (Figure 4,5).
- The presence of microsatellites in a given nucleotide sequence can easily be detected using the ‘Microsatellite Repeat Finder’ tool.
- The bulk data of all the markers are available for download.

## Conclusions

FmMDb holds a diverse range of DNA markers such as genomic-, genic-SSRs and ILP markers with unrestricted public access, thus bridging the gap between basic and applied sciences. Of note, the data presently made available at FmMDb has been solely generated in the author’s laboratory [5]–[9], and all the listed sequences were submitted to the NCBI probe database. Since our research team will be working on generating all possible genomic resources in foxtail millet, the database will be regularly updated. Moreover, the researchers who have developed molecular markers in foxtail millet with the aim of assisting the millet and bioenergy crop improvement are invited to submit their data to FmMDb. Hence, we hope that FmMDb will serve as a central resource for plant breeders as well as researchers who are dedicated towards crop improvement of millets and bioenergy grasses.
